# Advanced brain dopamine transporter imaging in mice using small-animal SPECT/CT

**DOI:** 10.1186/2191-219X-2-55

**Published:** 2012-09-29

**Authors:** Miia Pitkonen, Eero Hippeläinen, Mari Raki, Jaan-Olle Andressoo, Arto Urtti, Pekka T Männistö, Sauli Savolainen, Mart Saarma, Kim Bergström

**Affiliations:** 1 Centre for Drug Research, Faculty of Pharmacy, University of Helsinki, P.O. Box 56 (Viikinkaari 5E), Helsinki, 00014, Finland; 2 HUS Medical Imaging Center, Helsinki University Central Hospital, Helsinki, 00290, Finland; 3 Institute of Biotechnology, University of Helsinki, Helsinki, 00014, Finland; 4 Division of Pharmacology and Toxicology, Faculty of Pharmacy, University of Helsinki, Helsinki, 00014, Finland; 5 Department of Physics, University of Helsinki, Helsinki, 00014, Finland

**Keywords:** SPECT/CT, Dopamine transporters, ^123^I β-CIT, Tracer kinetics

## Abstract

**Background:**

Iodine-123-β-CIT, a single-photon emission computed tomography (SPECT) ligand for dopamine transporters (DATs), has been used for *in vivo* studies in humans, monkeys, and rats but has not yet been used extensively in mice. To validate the imaging and analysis methods for preclinical DAT imaging, wild-type healthy mice were scanned using ^123^I-β-CIT.

**Methods:**

The pharmacokinetics and reliability of ^123^I-β-CIT in mice (*n* = 8) were studied with a multipinhole SPECT/CT camera after intravenous injection of ^123^I-β-CIT (38 ± 3 MBq). Kinetic imaging of three mice was continued for 7 h postinjection to obtain the time-activity curves in the striatum and cerebellum volumes. Five mice had repeated measures 4 h post-^123^I-β-CIT injection to provide an indication of test-retest reliability. The same five mice served as a basis for a healthy mean SPECT template.

**Results:**

Specific binding of ^123^I-β-CIT within the mouse striatum could be clearly visualized with SPECT. The kinetics of ^123^I-β-CIT was similar to that in previously published autoradiography studies. Binding potential mean values of the test-retest studies were 6.6 ± 15.7% and 6.6 ± 4.6%, respectively, and the variability was 9%. The SPECT template was aggregated from the first and second imaging of the test-retest animals. No significant difference between the templates (*P* > 0.05) was found. From the test template, a striatal volume of 22.3 mm^3^ was defined.

**Conclusions:**

This study demonstrates that high-resolution SPECT/CT is capable of accurate, repeatable, and semiquantitative measurement of ^123^I-β-CIT DAT binding in the mouse brain. This methodology will enable further studies on DAT density and neuroprotective properties of drugs in mice.

## Background

Previously, sensitivity and resolution of the single-photon emission computed tomography (SPECT) system have been challenges in small-animal imaging. Currently, the scanning time can be kept short enough to enable dynamic acquisitions, due to the high level of detection efficiency and excellent spatial resolution obtained by multipinholes [1]. Here, we examined the feasibility of dual-modality-dedicated small-animal SPECT/computed tomography (CT) in submillimeter brain structures and the advantage of dynamic acquisitions.

Parkinson's disease (PD) is characterized by the progressive degeneration of nigrostriatal dopaminergic neurons. This neurodegenerative process is associated with a loss of striatal dopamine transporters (DATs) [2,3]. *In vivo* measurement of DAT density with SPECT is an early marker of dopaminergic cell loss in subjects with PD [4-6]. ^123^I-2β-carbomethoxy-3β-(4-iodophenyl)tropane (^123^I-β-CIT), a SPECT ligand for DATs, has not yet been validated in mice, which are frequently induced to model several diseases. Previous mouse studies are *ex vivo* autoradiography works [7-9], in which follow-up of the same animals was not possible and test-retest measurement was disabled. Whereas, Mochizuki et al. used a simple probe system offering only a single projection and therefore cannot differentiate between the brain structures [10]. However, the full benefit of small-animal imaging can only be achieved by *in vivo* quantification of radiotracers that enable the following of physiological processes over time in the same animal. In rats, such quantification has been performed using clinical gamma cameras [11,12]. The latest work was executed with a modern small-animal multipinhole SPECT by taking static images of the mouse brain [13]. In that work, the used DAT tracer was ^123^I-2β-carbomethoxy-3β-(4-iodophenyl)-*N*-(3-fluoropropyl)nortropane (FP-CIT; DatScan, GE Healthcare Inc., Waukesha, WI, USA), which is divergent from ^123^I-β-CIT, as will be discussed later in this chapter. The aim of our study was to determine the complimentary preclinical results since the kinetics between mice and rats may vary.

Absolute quantification of DATs in mice would require full kinetic modeling, with invasive arterial blood sampling and dynamic SPECT scanning. However, arterial blood sampling in mice is difficult, and other methods have been accepted for kinetic analysis [14,15]. For ^123^I-labeled tracers, good correlation has been demonstrated using the semiquantitative index of the specific DAT binding, referred to as binding potential (BP). BP is a ratio of the specific to nonspecific binding of DATs (such as the striatum and cerebellum). BP is directly proportional to the density of DATs in the equilibrium state [14,15]. We used BP to analyze the ^123^I-β-CIT ratios in the kinetics and test-retest studies of mice.

The ^123^I-β-CIT and FP-CIT are both clinically available and specific for use in PD diagnostics [16]. Here, we used ^123^I-β-CIT for several reasons: First, more rapid wash-up of the FP-CIT may cause underestimation of DAT density in patients [17]. Second, more rapid kinetics of FP-CIT in patients is even further accelerated in mice, which makes ^123^I-β-CIT more suitable for investigating disease models or pharmaceutical effects in mice. Third, with ^123^I-β-CIT, higher binding ratios have been reported in humans [17], which improve the signal-to-noise ratio in images acquired from the mouse brain.

To demonstrate the feasibility of the DAT tracer ^123^I-β-CIT in preclinical research in mice, we established the kinetics of the tracer with high-resolution SPECT/CT imaging. Furthermore, we validated the steady-state conditions of the tracer in the brain and evaluated the reproducibility of the method in test-retest measurements. Our second objective was to create a normal template to be used as a future reference frame for the coregistration and analysis of the mice with altered DAT production.

## Methods

### SPECT/CT system

SPECT/CT imaging was performed using a preclinical four-headed gamma camera with integrated CT system and dedicated multipinhole collimators, comprising in all 36 1.0-mm pinholes (nanoSPECT/CT, Bioscan Inc., Washington, DC, USA). The manufacturer states that the sensitivities of the pinhole collimators are >1,200 counts per second (cps)/MBq, with a maximum resolution of ≤0.75 mm. The scanning mode is helical for both modalities.

### Phantom studies

The spatial resolution of the system was verified for ^123^I, using a Jaszczak phantom with hot rods ranging from 0.7 to 1.2 mm (Bioscan Inc., Washington, D.C., USA). The phantom was filled with 75 MBq of ^123^I-NaI (MAP Medical Technologies Oy, Tikkakoski, Finland), and the images were acquired in 32 projections, using 150 s per gantry position. The imaged data were reconstructed with HiSPECT NG software (Scivis GmbH, Göttingen, Germany).

### Animals

The animal experiments were reviewed and approved by the Finnish National Animal Experiment Board and performed in accordance with Good Laboratory Practices for Animal Research. Triple-mixed genetic background 129Ola/C57BL/6/ICR mice were backcrossed once to the 129Ola line, and 4-month-old male progenies were used in the SPECT experiments. The animals were maintained under isoflurane inhalation anesthesia during radiotracer administration and imaging. Under anesthesia, body temperature of the animals was maintained using a heated animal bed (37°C) (Minerve, France), during imaging, and with a heating pad under the cage, between imaging sessions.

### SPECT/CT imaging

All mice (*n* = 8) received 34 to 41 MBq of ^123^I-β-CIT (MAP Medical Technologies Oy) in <200 μl intravenously. Kinetics study mice (*n* = 3) received 34 to 41 MBq of ^123^I-β-CIT. The test-retest mice received 1.12 ± 0.08 MBq/g body weight (b.wt.) and 0.94 ± 0.07 MBq/g b.wt., respectively.

At the early time points (from 20 min to 2 h 30 min) of the kinetics experiments (*n* = 3), the data were acquired dynamically with a scanning length of 15 min (angular step 15°, 24 projections, 150 s per gantry position). At the late time points (at 4 h and 7 h), the scanning length was 25 min (angular step 15°, 24 projections, 250 s per gantry position). In all, 11 time points were collected from the mice. The data were acquired in a 128 × 128 matrix with pixel size and a slice thickness of 0.2 mm.

CT was acquired at 20 min and later at 4 and 7 h. The CT parameters used in the present work were as follows: 180 projections, pixel size of 192 × 192 μm, X-ray source of 45 kVp, and exposure time of 500 ms. Helical scanning is used by both modalities and is performed by moving the animal through the SPECT and CT.

In the test-retest experiment (*n* = 5), the mice were imaged 4 h postinjection, which was confirmed to be the equilibrium time point in the kinetics study. The same animals went through the same protocol after 8 days. The same SPECT protocol was used as described for the late time points in the kinetics experiment.

### Data analysis: kinetics experiment

The data were reconstructed in the system's dedicated reconstruction program with an iterative reconstruction algorithm, resulting in a voxel size of 0.3 mm. After image reconstruction, the images were straightened and analyzed using InVivoScope software (Bioscan Inc.). Straightening of the images was assisted by the CT images. To avoid the variability of the slice selection and to gain statistical power, we used the entire striatum volume for the analysis. Time-activity curves were obtained from the kinetics data by manually delineating the volumes of interest (VOIs). At each time point, the VOIs were drawn over specific (striatal) and nonspecific (cerebellar) brain structures, and the mean counts in these two areas were measured against time. From the mean values obtained, we calculated the BP, which represents the ratio of the distribution volumes of the specifically and the nonspecifically bound compartment.

### Data analysis: test-retest experiment

Following data reconstruction, the images were first manually straightened and converted from Dicom to the Analyze format [18] and further processed in Vinci (S. Vollmar et al., Cologne, Germany) [19]. In Vinci, the images were first masked to remove the eyes and other possible high-uptake areas external to the brain. Then, all the images were normalized individually using the average value of the voxels within 95% of the maximum count value in the striatal volume. Finally, both time points were registered to the SPECT template (see below), which served as a reference space and defined the VOIs. The mean count density per pixel in each region was calculated and corrected for the effects of decay. The test-retest variability was calculated as the absolute difference of two measurements divided by the mean of two measurements as a percentage. Comparison between the test and retest time points was achieved by the Wilcoxon signed rank test.

### Creation of template

Two separate templates were aggregated from the test and retest mice groups. The images were first manually straightened, masked, and normalized to 95% of the maximum count value. Secondly, the images were spatially coregistered using an affine transformation algorithm with mutual information as a similarity measure for registrations. Then, the average and standard deviation templates were calculated using the spatial- and count-normalized images. Finally, the anatomical areas were confirmed by coregistering the SPECT template to an available magnetic resonance imaging (MRI) brain template [20]. Comparison between the test and retest templates was achieved by voxel-by-voxel *t* test.

## Results

### SPECT imaging of phantom

**Figure 1:**
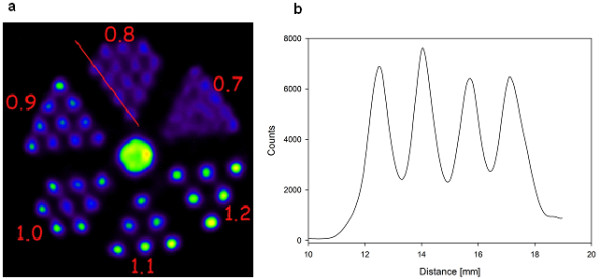
**Jaszczak phantom.** The spatial resolution of the system was verified for ^123^I, using a Jaszczak phantom with hot rods ranging from 0.7 to 1.2 mm. Images were acquired in 32 projections, using 150 s per gantry position. Jaszczak phantom, filled with 75 MBq of ^123^I-Na, demonstrates the spatial resolution of the system. The red line in the SPECT image (**a**) represents the area where the count profile curve (**b**) is captured. The 0.8-mm hot rods are clearly detectable.

Prior to the animal studies, we evaluated the performance of the scanner for spatial resolution. Under the conditions of high radioactivity (75 MBq), acquisition time (20 min), and reconstruction parameters for fine image quality, the resolution was superior. Figure 1 shows the SPECT images and their count profile curves of the Jaszczak phantom filled with ^123^I-Na, yielding an excellent spatial resolution with good visualization of hot rods as thin as 0.8 mm.

### SPECT template

**Figure 2:**
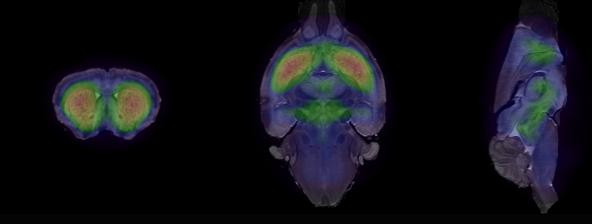
**Coregistered template.** In building the template, the anatomical areas are confirmed by coregistering the template to an available MRI brain template [20]. Coregistration is visualized so that SPECT is in color and MRI is in gray scale.

Templates were compared using voxel-by-voxel *t* test, and no significant differences were found between test and retest. Figure 2 shows the outcome of coregistration between the created SPECT template and MRI template. The anatomical areas seem to be equivalent between SPECT and MRI (Figure 2).

**Figure 3:**
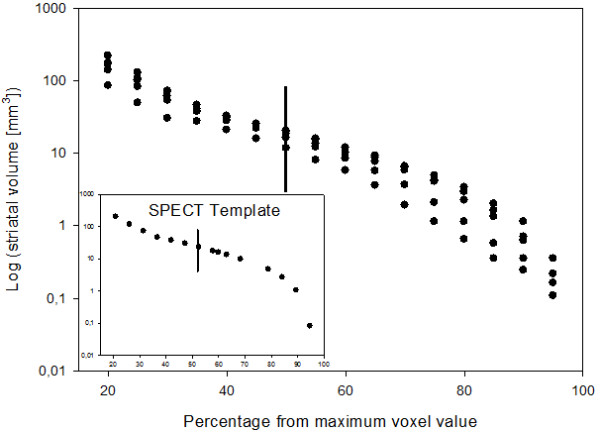
**Striatal volumes.** Mice (*n* = 5) received 1.12 ± 0.08 MBq/g b.wt. of ^123^I-β-CIT. They were imaged 4 h postinjection, and scanning length was 25 min (angular step 15°, 24 projections, 250 s per gantry position). The data were acquired in a 128 × 128 matrix with pixel size and a slice thickness of 0.2 mm. The anatomical volume of the striatum was estimated by several thresholds from the maximum intensity of the structure. A striatal volume was measured for the template and individual mice by choosing a threshold of 50% of the maximum intensity of the striatum (vertical line), which resulted as a striatal volume of 22.3 mm^3^ and 17.2 mm^3^ ± 20%, respectively.

In the individual animals and the template, the anatomical volume of the striatum was estimated by several thresholds from the maximum intensity of the structure (Figure 3). From the template, we obtained a striatal volume of approximately 22.3 mm^3^ by choosing a threshold of 50% of the maximum intensity of the striatum (Figure 3). When performing the same analysis for individual animals, we obtained a striatal volume of 17.2 mm^3^ ± 20% (Figure 3).

Dopaminergic terminals are present at high density in the striatum but are less dense in the cerebellum, which makes delineation of the nonspecific uptake area very difficult. Thus, the cerebellar VOI was chosen to consist of only part of the cerebellum (13.3 mm^3^) to avoid delineation of those areas that consist of counts due to scattered or partial volume effects. Meanwhile, the cerebellar volume should be sufficiently large to gain enough statistics for calculation of a reliable BP.

### Kinetics study of mice

**Figure 4:**
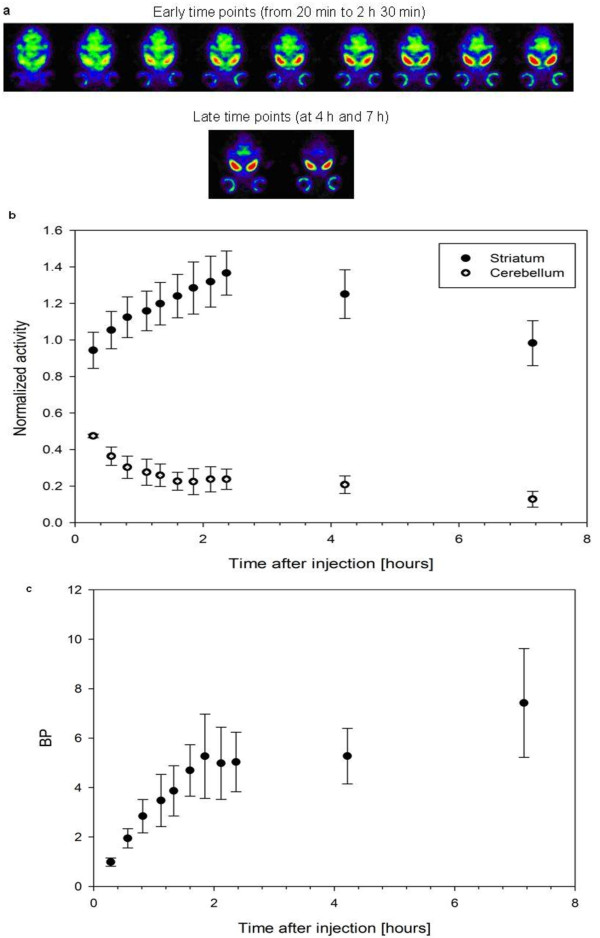
**Kinetics experiment data.** Mice (*n* = 8) received 34 to 41 MBq of ^123^I-β-CIT. At the early time points (from 20 min to 2 h 30 min) post-tracer injection, the data were acquired dynamically with a scanning length of 15 min (angular step 15°, 24 projections, 150 s per gantry position). At the late time points (at 4 h and 7 h), the scanning length was 25 min (angular step 15°, 24 projections, 250 s per gantry position). The data were acquired in a 128 × 128 matrix with pixel size and a slice thickness of 0.2 mm. In all, 11 time points were imaged (**a**). Time-activity curves were obtained from the kinetic data by manually delineating the VOI (**b**). At each time point, the VOIs were drawn over specific (striatal) and nonspecific (cerebellar) brain structures, and the mean counts in these two areas were measured against time. Specific-to-nonspecific BP was calculated (**c**).

In the kinetics experiment, a total of 11 time points were imaged, starting at 20 min and ending at 7 h postinjection (Figure 4a). In the striatum, the amount of activity increased gradually and peaked at approximately 2 to 3 h (Figure 4b). After 4 h, the striatum uptake washed out gradually. The nonspecific binding area, the cerebellum, showed no binding and half of the activity dissipated in 2.5 h. The ratio of the specific binding value of ^123^I-β-CIT to nonspecific uptake, BP, became constant 2 h postinjection (Figure 4c). After 4 h, BP shows a gradual increase. Symmetrical striatal binding was visible in the SPECT images at 4 h postinjection (Figure 4a). Harder's glands showed radioactivity uptake (Figure 4a).

### Test-retest study

**Figure 5:**
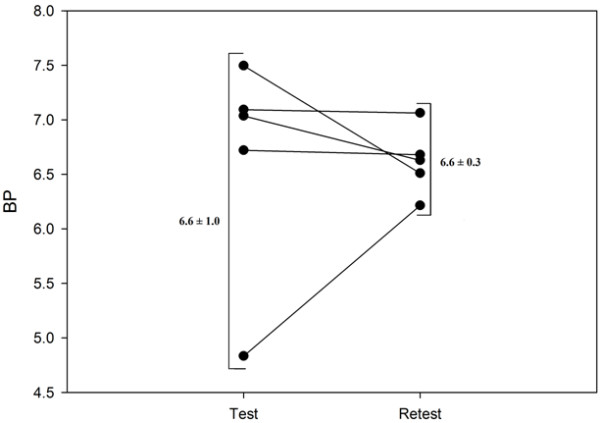
**Binding potential values.** The test and retest mice (*n* = 5) received 1.12 ± 0.08 MBq/g b.wt. and 0.94 ± 0.07 MBq/g b.wt. of ^123^I-β-CIT, respectively. Mice were imaged with the equivalent protocol in the test and retest time points; scanning length was 25 min (angular step 15°, 24 projections, 250 s per gantry position). The data were acquired in a 128 × 128 matrix with pixel size and a slice thickness of 0.2 mm. Individual BP values were obtained at 4 h postinjection on each of the five mice at both test and retest (8 days later), with the corresponding mean and standard deviation. The test-retest measurements were insignificantly different (*P* = 0.968).

In the test-retest study, the 4-h time point was set as the acquisition time, based on the kinetics experiment. The same animals followed the same protocol after 8 days. The individual BP values obtained from each mouse are shown in Figure 5. The mean BP values of the test-retest studies were 6.6 ± 15.7% and 6.6 ± 4.6% (mean ± relative standard error), respectively, and these measures did not differ statistically from each other (*P* = 0.968). The test-retest variability was 9%.

## Discussion

The primary aim of this study was to demonstrate that small-animal SPECT/CT imaging is capable of measuring DAT density. We validated a DAT-binding imaging protocol for the mouse brain, using ^123^I-β-CIT. To our knowledge, the present study is the first to demonstrate the 3D kinetics of ^123^I-β-CIT in mice, using SPECT/CT imaging.

The SPECT/CT used was equipped with four collimators and apertures, each with nine 1-mm pinholes, resulting in high counting rates. The resolution for the camera was in the submillimeter range; Jaszczak phantom imaging showed that we can reliably image the mouse striatum.

From the template, we obtained a striatal volume of approximately 22.3 mm^3^ with a threshold of 50% of the maximum intensity of the striatum. Similar striatal volumes have been reported in equivalent strains [21]. At the same threshold, we obtained a striatal volume of 17.2 mm^3^ ± 20%. In Figure 3, the striatal volume is shown as a function of thresholds in individuals and the template. The striatal volume increases until it becomes stable at approximately 50% to 60% of the maximum activity. The phenomenon was found in both cases (Figure 3). For threshold values higher than 60%, the volume spreads outside the striatal volumes, into other parts of the brain. The higher striatal volume in the template is due to individual variation, coregistration, and averaging. This comparison indicates that the template represents data in healthy mice well and that no coregistration or technical problems occurred. Further, we compared the templates created from the first and second imaging of the test-retest study and found no significant difference. This result demonstrates that our method has good repeatability. In future works, we will address individual variation in striatal structures and minimize the processing steps needed using the template.

In the present study, we report the kinetics of the ^123^I-β-CIT tracer in the striatum and cerebellum. As indicated earlier, the quantitative validity of the BP method (striatal to background brain region ratio) depends on the achievement of steady levels of activity in these regions [17]. As shown in Figure 4b, 3 h postinjection, there is parallel washout in cerebellum and striatal volumes. Thus, BP analysis can be considered valid 4 h post-^123^I-β-CIT injection in the mouse brain. These results agree with the earlier autoradiography works [7-9]. However, our results show moderate increase in BP, which is evident at the 7-h imaging point. This might be due to the individual kinetics of mice and also because of low statistics in the cerebellum volume. To resolve this, further kinetic studies should be performed at late imaging points.

We used BP to perform semiquantitative analysis of dopaminergic neurotransmission post-^123^I-β-CIT injection. BP increased over the time period followed, similar to previous rat studies with SPECT [11,12] and autoradiography work with both rats [22] and mice [7-9]. In healthy human subjects, the evolvement of BP is similar, differing only by time scale [23,24], due to the slower metabolism of humans compared with mice. In the present work, 4 h postinjection was a suitable time point for use in the test-retest study, which is equivalent to 20 to 24 h in human patients [23,24].

In the test-retest study, one of the five animals showed a clearly larger variation among BPs, which probably resulted from unsuccessful injection at either one of the time points. The variability between test and retest was clearly better than that in healthy human subjects [25,26], which shows that our method has good test-retest reproducibility. Such improvement was expected, due to the better sensitivity and resolution of the small-animal SPECT instrument compared with clinical systems.

Successful validation of the imaging protocol and establishment of the template will help to reduce the number of control mice needed for the experiments and will further reduce the overall costs and time needed. The template created here will be further used as a reference to differentiate between normal and genetically modified mouse DAT densities. Also, we are planning to extend the template to include serotonin transporters. In present and future works, the template will offer a reference for coregistration and will take into account the variance between individuals in the VOI analysis. Furthermore, correct and repeatable delineation of VOIs is needed to gain enough statistical power because the targets are submillimeter in size. Herein, we focused on certain VOIs, but the template may also be used in voxel-by-voxel analysis.

## Conclusions

We have demonstrated explicitly that high-resolution, multipinhole SPECT/CT of mice is capable of accurate, repeatable, and semiquantitative measurement of DAT binding, using ^123^I-β-CIT. This methodology should increase the opportunities for further study of cerebral binding sites, especially in mice.

## Competing interests

The authors declare that they have no competing interests.

## Authors' contributions

MP, EH, and MR carried out the SPECT/CT experiments, participated in the design of the study, and drafted the manuscript. MP and EH carried out the data analysis and template creation. J-OA and MS involved in conceiving the study and provided the animals. AU, PTM, and SS were involved in conceiving the study and revising the manuscript. KB conceived the study, participated in its design and coordination, and helped draft the manuscript. All authors read and approved the final manuscript.

## References

[B1] Acton PD Choi SR Plossl K Kung HF Quantification of dopamine transporters in the mouse brain using ultra-high resolution single-photon emission tomography Eur J Nucl Med Mol Imaging 2002 29 691 698 10.1007/s00259-002-0776-7 11976810

[B2] Kaufman MJ Madras BK Severe depletion of cocaine recognition sites associated with the dopamine transporter in Parkinson's-diseased striatum Synapse 1991 9 43 49 10.1002/syn.890090107 1796351

[B3] Niznik HB Fogel EF Fassos FF Seeman P The dopamine transporter is absent in parkinsonian putamen and reduced in the caudate nucleus J Neurochem 1991 56 192 198 10.1111/j.1471-4159.1991.tb02580.x 1987318

[B4] Guttman M Burkholder J Kish SJ Hussey D Wilson A DaSilva J Houle S [11C]RTI-32 PET studies of the dopamine transporter in early dopa-naive Parkinson's disease: implications for the symptomatic threshold Neurol 1997 48 1578 1583 10.1212/WNL.48.6.1578 9191769

[B5] Winogrodzka A Bergmans P Booij J van Royen EA Janssen AG Wolters EC [123I]FP-CIT SPECT is a useful method to monitor the rate of dopaminergic degeneration in early-stage Parkinson's disease J Neural Transm 2001 108 1011 1019 10.1007/s007020170019 11716136

[B6] Tissingh G Booij J Bergmans P Winogrodzka A Janssen AG van Royen EA Stoof JC Wolters EC Iodine-123-N-omega-fluoropropyl-2beta-carbomethoxy-3beta-(4-iod ophenyl)tropane SPECT in healthy controls and early-stage, drug-naive Parkinson's disease J Nucl Med 1998 39 1143 1148 9669384

[B7] Cline EJ Scheffel U Boja JW Mitchell WM Carroll FI Abraham P Lewin AH Kuhar MJ In vivo binding of [125I]RTI-55 to dopamine transporters: pharmacology and regional distribution with autoradiography Synapse 1992 12 37 46 10.1002/syn.890120105 1411962

[B8] Scheffel U Dannals RF Cline EJ Ricaurte GA Carroll FI Abraham P Lewin AH Kuhar MJ [123/125I]RTI-55, an in vivo label for the serotonin transporter Synapse 1992 11 134 139 10.1002/syn.890110206 1385663

[B9] Boja JW Mitchell WM Patel A Kopajtic TA Carroll FI Lewin AH Abraham P Kuhar MJ High-affinity binding of [125I]RTI-55 to dopamine and serotonin transporters in rat brain Synapse 1992 12 27 36 10.1002/syn.890120104 1411961

[B10] Mochizuki T Mochizuki T Villemagne VL Scheffel U Liu X Musachio JL Dannals RF Wagner HN Jr A simple probe measures the pharmacokinetics of [125I]RTI-55 in mouse brain in vivo Eur J Pharmacol 1997 338 17 23 10.1016/S0014-2999(97)01307-1 9407999

[B11] Scherfler C Donnemiller E Schocke M Dierkes K Decristoforo C Oberladstatter M Kolbitsch C Zschiegner F Riccabona G Poewe W Wenning G Evaluation of striatal dopamine transporter function in rats by in vivo beta-[123I]-CIT pinhole SPECT Neuroimage 2002 17 128 141 10.1006/nimg.2002.1158 12482072

[B12] Laruelle M Baldwin RM Malison RT Zea-Ponce Y Zoghbi SS al-Tikriti MS Sybirska EH Zimmermann RC Wisniewski G Neumeyer JL SPECT imaging of dopamine and serotonin transporters with [123I]beta-CIT: pharmacological characterization of brain uptake in nonhuman primates Synapse 1993 13 295 309 10.1002/syn.890130402 7683143

[B13] Pissarek MB Oros-Peusquens AM Schramm NU Challenge by the murine brain: multi-pinhole SPECT of 123I-labelled pharmaceuticals J Neurosci Methods 2008 168 282 292 10.1016/j.jneumeth.2007.10.011 18061274

[B14] Laruelle M Giddings SS Zea-Ponce Y Charney DS Neumeyer JL Baldwin RM Innis RB Methyl 3 beta-(4-[125I]iodophenyl)tropane-2 beta-carboxylate in vitro binding to dopamine and serotonin transporters under "physiological" conditions J Neurochem 1994 62 978 986 8113817 10.1046/j.1471-4159.1994.62030978.x

[B15] Abi-Dargham A Gandelman MS DeErausquin GA Zea-Ponce Y Zoghbi SS Baldwin RM Laruelle M Charney DS Hoffer PB Neumeyer JL Innis RB SPECT imaging of dopamine transporters in human brain with iodine-123-fluoroalkyl analogs of beta-CIT J Nucl Med 1996 37 1129 1133 8965183

[B16] Kuikka JT Bergstrom KA Ahonen A Hiltunen J Haukka J Lansimies E Wang S Neumeyer JL Comparison of iodine-123 labelled 2 beta-carbomethoxy-3 beta-(4-iodophenyl)tropane and 2 beta-carbomethoxy-3 beta-(4-iodophenyl)-N-(3-fluoropropyl)nortropane for imaging of the dopamine transporter in the living human brain Eur J Nucl Med 1995 22 356 360 10.1007/BF00941854 7607268

[B17] Seibyl JP Marek K Sheff K Zoghbi S Baldwin RM Charney DS van Dyck CH Innis RB Iodine-123-beta-CIT and iodine-123-FPCIT SPECT measurement of dopamine transporters in healthy subjects and Parkinson's patients J Nucl Med 1998 39 1500 1508 9744331

[B18] Nolf E XMedCon - an open-source medical image conversion toolkit 1991 http://xmedcon.sourceforge.net/

[B19] Vollmar S Sué M Krais R Hohmann C Hüsgen A Max Planck Institute for Neurological Research (Cologne. Germany: Vinci 3) http://www.nf.mpg.de/vinci3/

[B20] Johnson GA Ali-Sharief A Badea A Brandenburg J Cofer G Fubara B Gewalt S Hedlund LW Upchurch L High-throughput morphologic phenotyping of the mouse brain with magnetic resonance histology Neuroimage 2007 37 82 89 10.1016/j.neuroimage.2007.05.013 17574443 PMC1994723

[B21] Rosen GD Williams RW Complex trait analysis of the mouse striatum: independent QTLs modulate volume and neuron number BMC Neurosci 2001 2 5 10.1186/1471-2202-2-5 11319941 PMC31432

[B22] Fujita M Takatoku K Matoba Y Nishiura M Kobayashi K Inoue O Nishimura T Differential kinetics of [123I]beta-CIT binding to dopamine and serotonin transporters Eur J Nucl Med 1996 23 431 436 10.1007/BF01247372 8612664

[B23] Laruelle M Wallace E Seibyl JP Baldwin RM Zea-Ponce Y Zoghbi SS Neumeyer JL Charney DS Hoffer PB Innis RB Graphical, kinetic, and equilibrium analysis of in vivo [123I]beta-CIT binding to dopamine transporters in healthy human subjects J Cereb Blood Flow Metab 1994 14 982 994 10.1038/jcbfm.1994.131 7929662

[B24] Kim SE Choi JY Choe YS Lee WY Serotonin transporters in the midbrain of Parkinson's disease patients: a study with [123I]beta-CIT SPECT J Nucl Med 2003 44 870 876 12791812

[B25] Seibyl JP Marek K Sheff K Baldwin RM Zoghbi S Zea-Ponce Y Charney DS van Dyck CH Hoffer PB Innis RB Test/retest reproducibility of iodine-123-betaCIT SPECT brain measurement of dopamine transporters in Parkinson's patients J Nucl Med 1997 38 1453 1459 9293807

[B26] Seibyl JP Laruelle M van Dyck CH Wallace E Baldwin RM Zoghbi S Zea-Ponce Y Neumeyer JL Charney DS Hoffer PB Innis RB Reproducibility of iodine-123-beta-CIT SPECT brain measurement of dopamine transporters J Nucl Med 1996 37 222 228 8667048

